# Incidence and Risk Factors for Surgical Site Infections Following Oromaxillofacial Oncologic Surgery in Dogs

**DOI:** 10.3389/fvets.2021.760628

**Published:** 2021-10-18

**Authors:** Brittney E. Rigby, Kevin Malott, Scott J. Hetzel, Jason W. Soukup

**Affiliations:** ^1^Department of Surgical Science, School of Veterinary Medicine, University of Wisconsin, Madison, WI, United States; ^2^Department of Biostatistics and Medical Informatics, University of Wisconsin, Madison, WI, United States

**Keywords:** oromaxillofacial surgery, surgical site infection, oncologic surgery, dog, antibiotic stewardship

## Abstract

Antibiotic stewardship in veterinary medicine is essential to help prevent resistant bacterial infections. Critical evaluation into the benefits of prophylactic use of antibiotics during veterinary surgical procedures is under reported and additional investigation is warranted. The objectives of this paper were to determine the incidence of surgical site infection in dogs that underwent oromaxillofacial oncologic surgery and to identify risk factors for the development of surgical site infection. In this retrospective cohort study including 226 dogs surgically treated for oromaxillofacial tumors between January 1, 1997 and December 31, 2018, the incidence of surgical site infection was determined to be 7.5%. Univariable logistical regression models were used to evaluate potential risk factors for development of surgical site infections including signalment, tumor type, antibiotic protocol, time under anesthesia, location of surgical procedure (dental suite vs. sterile operating room), specific comorbidities, and surgical margins obtained. Anesthetic events lasting greater than 6 h were significantly associated with development of infection. Signalment, comorbidities, administration of anti-inflammatory and immunosuppressive medications, tumor type, histological margin evaluation, surgical procedure location, and antibiotic protocols were not significant contributors to development of infection. Use of antibiotic therapy in this cohort was not protective against development of infection and may not be routinely indicated for all oromaxillofacial oncologic surgeries despite common promotion of its use and the contaminated nature of the oral cavity. Anesthetic time significantly contributed towards the development of infection and use of perioperative antibiotics for surgical procedures lasting >6 h may be routinely warranted.

## Introduction

A surgical site infection is defined by the Centers for Disease Control and Prevention (CDC) as a post-surgical infection that occurs within 30 days of the surgical procedure (or within 1 year of an implant placement) and must include at least one of the following features: purulent debris; positive bacterial culture; or pain, swelling, heat, and redness at the surgical site (1). Surgical site infection rates have been directly correlated with the degree of contamination of the surgical site. More specifically, infection rates in cats and dogs range from 2.0–4.8%, 3.5–5.0%, 4.6–12.0%, and 6.7–18.1% in clean, clean-contaminated, contaminated, and dirty surgical procedures, respectively (2–4). Few reports describing veterinary oromaxillofacial surgery report information regarding the incidence of surgical site infections, however, when included, the rate in veterinary patients has been reported as 0–7.9% (2–4).

Peri-operative antibiotics have been recommended for prevention of surgical site infections and used routinely for various surgical procedures in both human and veterinary medicine (5–8). With growing concern for antibiotic resistant bacteria and infections, critical analysis of the appropriate use of antibiotics in veterinary medicine is necessary. Consensus among veterinarians on appropriate use of prophylactic antibiotic therapy does not exist. The American Veterinary Dental College (AVDC) position statement recommends systemically administered antibiotics to reduce bacteremia in only a small subset of animals with underlying systemic disease, such as sub-aortic stenosis or severe renal disease, and in the face of a severe oral infection (9). The position statement addresses systemic antibiotic use but does not differentiate between prophylactic and therapeutic treatment. The American Dental Association (ADA) recommends prophylactic antibiotic therapy for a very small population including patients with infective endocarditis and patients with a history of complications following joint replacement (10). Both the AVDC and ADA statements are generalized to “dentistry procedures” and do not address the difference between oncologic procedures which carry higher consequences of complications when compared to simple dental extractions. The American Animal Hospital Association (AAHA) has detailed guidelines regarding appropriate use of antibiotics in veterinary medicine, however, none apply to the unique surgical environment of the orofacial region (11). For example, the AAHA guidelines recommend performing culture and sensitivity to determine appropriate antibiotic selection for all clean-contaminated, contaminated, and dirty surgical procedures. However, this seems an impractical when considering surgery involving the ever-contaminated oral cavity. These guidelines state, “The use of antimicrobials to prevent infection can only be justified in cases where bacterial infection is likely to occur,” but do not define the recommendation further (11). While the degree of contamination is considered significant risk for surgical site infection, other variables have also been identified as risk factors. Risk factors previously identified include patient signalment, time under anesthesia, surgical time, specific patient comorbidities and American Society of Anesthesiologist (ASA) status, and complications under anesthesia (2, 12–14).

There are two circumstances in which prophylactic antibiotic therapy is generally recommended. First, prophylactic antibiotic therapy may be recommended when catastrophic consequences of a surgical site infection, such as implant failure or bacterial peritonitis following gastrointestinal surgery, are of concern. Second, when the risk of infection is relatively high. Since the degree of contamination during a surgical procedure is considered to be a large contributing factor to the development of infection, those procedures which are not “clean” are considered high risk for infection and prophylactic antibiotic therapy has been advocated (15). Surgical access through the oral cavity is often necessary for oromaxillofacial oncologic surgery, thus, these procedures are generally considered clean-contaminated, contaminated, or dirty as the oral cavity has high loads of bacterial contamination. Oral neoplasms also often have devitalized or necrotic tissue which may alter the contaminated status of the surgical field. Additionally, surgical site infections following oromaxillofacial oncologic surgery can result in significant complications, such as dehiscence resulting in oronasal fistula formation following a maxillectomy (16–19).

Although the majority of published material state that clean-contaminated, contaminated, and dirty procedures warrant prophylactic use of antibiotics, there is little evidence to prove its benefit, especially in regard to oromaxillofacial surgery (2, 12). Antibiotic administration carries risk of undesirable side effects for the patient that should not be overlooked, such as gastrointestinal distress and allergic reaction. These unwanted consequences may lead to unnecessary cost to the client and a prolonged hospital stay for the patient (14). Additionally, inappropriate use of antibiotics can contribute to development of bacterial resistance and antibiotic resistant infections, which is a serious public health issue (20–22). Thus, investigation into the usefulness of peri-or post-operative antibiotics for these procedures is indicated.

The purpose of this retrospective study was to determine if the use of prophylactic antibiotic therapy resulted in decreased incidence in surgical site infections following oromaxillofacial oncologic surgery. A secondary aim of this investigation was to identify factors that may predispose patients to be at an increased risk for surgical site infection. Our hypothesis was that oromaxillofacial oncologic surgeries would have surgical site infection rates consistent with a contaminated surgical field (4.6–12%). We also hypothesized that there would be no significant difference in the incidence of surgical site infection when considering the antibiotic protocol, tumor type, or histologic margin assessment.

## Methods

The following search terms were used to identify the medical records of patients that underwent maxillofacial surgery at this institution between January 1, 1997 and December 31, 2018: mandibulectomy, maxillectomy, zygomatectomy, coronoidectomy, oronasal fistula, cleft palate, orbitectomy, incisivectomy, piezosurgery, condylectomy, rim excision, and nasal planectomy. Eight-hundred and eighty patients were identified using these search terms. These 880 records were further reviewed to identify those that underwent surgical treatment of oromaxillofacial tumors. Three hundred and seventy-five cases were identified. Only surgeries with the aim of achieving tumor-free surgical margins were included. Medical records describing palliative-intent surgical treatment or anticipation of incomplete excision on histologic review were excluded. There was wide variability in the pre-surgical diagnostic imaging that each patient received ranging from no imaging, dental radiographs only, skull radiographs, and head computed tomography (CT). In attempt to standardize the case selection, only those cases which had pre-operative head CT for diagnostic and surgical planning were included in this report. Cases that did not have a head CT were excluded. Case inclusion required accessibility to information about the patient's medical history, physical examination at the time of the surgery, surgical report, hospitalization summary, anesthetic record, and histology report. A description of a post-surgical recheck at least 7 days after the surgical procedure at this institution or with the referring veterinarian was also required for inclusion.

A surgical site infection was defined by criteria outlined by the CDC. The criteria define that an infection must occur within 30 days of the surgical procedure (or within 1 year of an implant placement) and have at least one of the following criteria: purulent discharge; positive bacterial culture; or pain, tenderness, swelling, redness, or heat. Using this definition, if the medical records describing the post-operative recheck(s) overtly stated the patient was diagnosed with a surgical site infection, had purulent discharge, included a positive bacterial culture, or included a description of pain, tenderness, swelling, redness, and / or heat, the patient was considered to have an infection in the statistical analysis. Numerous risk factors were analyzed as variables for development of surgical site infection in this population. Variables analyzed included patient signalment, specific comorbidities, use of immunosuppressive or non-steroidal anti-inflammatory medications at the time of surgery, tumor type, histologic margin assessment at the time of excision, location of the surgical procedure, and the antibiotic protocol used.

The patient signalment including age at time of surgery, sex, and weight of each patient was recorded. Diagnosis of an endocrinopathy was identified. Chronic or current use of immunosuppressive medications and non-steroidal anti-inflammatory medications was analyzed. Time under anesthesia was analyzed as a variable, however, surgical time was not consistently available in the medical records and, therefore, not evaluated. Tumors were classified as malignant or odontogenic based on the final histopathologic diagnosis of the excised oromaxillofacial mass, when available.

Histopathologic margins were identified using the final histopathology report from the time of the surgical procedure and defined as simply tumor-free margins or incomplete excision for the purpose of this study. Margins were considered tumor-free if the pathologist reported seeing no neoplastic cells at the edge of the submitted sample. Tumor classification and achievement of tumor-free vs. incompletely excised margins was considered in the statistical analysis as a potential risk for infection development. Surgical closure of gross neoplastic tissue often results in poor healing, potentially increasing the risk of infection. The authors included the histopathologic surgical margins in this statistical analysis to identify if microscopic disease was a risk factor for development of surgical site infection.

Finally, administration of antibiotics was considered. For the purposes of this study, antibiotic use was divided into four categories including pre-operative, peri-operative, post-operative, or no antibiotic administration. Pre-operative antibiotics were defined as oral antibiotics given to the patient within the 7 days immediately leading up to the surgery. Peri-operative antibiotic use was defined as administration of antibiotics (oral or intravenous) in the hospital no earlier than 2 h pre-procedure and continued no later than 24 h after the procedure. While oral administration of antibiotics is not the recommended route for surgical prophylaxis, patients who were administered oral antibiotics within 2 h of their procedure were considered to have received peri-operative antibiotics to distinguish them from those patients who were receiving pre-operative antibiotics but not within the 2 h time period immediately before the surgical procedure. Post-operative administration included any antibiotics administered more than 24 h following the end of surgery or dispensed for administration at home. Patients that did not receive any pre-operative, peri-operative, or post-operative antibiotics as defined above were categorized as having no antibiotic treatment. Antibiotics that were prescribed at a post-surgical recheck appointment, but were not dispensed at the time of discharge, were not considered in this analysis.

## Statistical Analysis

Descriptive statistics were used to report patient signalment including age at time of surgery, sex, and weight and to report specific patient comorbidities and medication use. The relationship between the variables and the development of a surgical site infection was evaluated using univariable logistical regression models. Results of the logistic regression models are presented as odds ratios (OR) with 95% confidence intervals (CI) and *p*-values. *Post-hoc* comparison of significant three-level factors utilized a Holm adjustment for three tests. All tests were conducted at a standard 5% significance level. Statistical calculations were made with R version 3.5 software.

## Results

The medical records search identified 375 dogs that underwent definitive-intent surgical excision of oromaxillofacial tumors. Two hundred twenty-six dogs met the inclusion criteria and were included in this report. There were 2 (0.9%) intact females, 103 (45.6%) spayed females, 15 (6.6%) intact males, and 106 (47%) castrated male dogs. The mean weight was 29.2kg (3.4–71kg) and mean age was 8.2 years old (7 months – 16 years old).

Out of 226 cases included in this analysis, 17 (7.5%) developed a surgical site infection. Nine out of 105 (8.6%) female dogs and eight out of 121 (6.6%) male dogs developed surgical site infections, with no statistically significant difference in sex and the development of infection (OR [95% CI]: 0.76 [0.28–2.03]; *p* = 0.578). Neither weight nor age were significantly associated with surgical site infection. Hypothyroidism was the only described endocrinopathy (*n* = 2) and there was no statistically significant association between the development of surgical site infection in these patients. Due to the low patient population receiving NSAID or immunosuppressive medications, these patients were analyzed together to provide more statistical strength. Thirteen patients were receiving either a NSAID medication or immunosuppressive medication at the time of the surgery. These medications included carprofen, meloxicam, deracoxib, azathioprine, and prednisone. There was no significant association between the development of infection in these patients.

The time under anesthesia ranged from 1.25 h to 10 h. The range was due to the varied surgical procedures ranging from mandibular rim excision or incisivectomy for treatment of benign tumors to excision of highly invasive malignant tumors with en bloc excision of the orbitozygomaticomaxillary complex with regional lymph node extirpation. The mean (SD) length of anesthesia was 4.4 (1.4) h for patients that did not develop infection vs. 4.9 (1.8) h for patients that did develop infections (*p* = 0.179). Time under anesthesia was initially subdivided into four categories of 0–2 h, 2–4 h, 4–6 h, and >6 h, however, with only two patients under anesthesia for 0–2 h, the former two categories were combined to include all patients anesthetized for <4 h. Time under anesthesia was subdivided into three categories of those lasting from 0–4 h (*n* = 100), 4–6 h (*n* = 92), and greater than 6 h (*n* = 31). Anesthetic time was significantly associated with rate of infection within these groups (ANOVA *p* = 0.048). *Post-hoc* analysis found that those procedures lasting 4–6 h were significantly less likely to develop infection as compared to procedures lasting greater than 6 h (*p* = 0.048). Although, when specifically compared to the 7.0% infection rate in procedures lasting 0–4 h, procedures lasting 4–6 h [4.3% infected, 95% CI; OR 0.6 (0.17–2.13); *p* = 0.541] and procedures lasting longer than 6 h [19.4% infected, 95% CI; OR 3.19 (0.98–10.34); *p* = 0.156] were not significantly more or less likely to develop infection.

One hundred and thirty nine tumors were malignant, 74 odontogenic and seven non-neoplastic upon final histologic analysis. Histopathologic samples were non-diagnostic in three cases, and there were three cases with a combination of odontogenic and malignant tumor identified within the submitted sample. Of the patients with malignant tumors, 11 (7.9%) developed infections. Six (8.1%) patients with odontogenic tumors developed infections. The difference in infection rates between patients with malignant and odontogenic tumors was not statistically significant. Margins were not reported in 16 out of the 226 cases (7.1%). This left 197 cases that could be evaluated for margins. Margins free of neoplastic cells were reported in 156 (79.2%) cases. Forty-one (20.8%) cases had incomplete margins. Infection was reported in 12 (7.7%) patients with tumor-free margins and in four (9.8%) patients with incompletely excised margins, a difference that was not statistically significant (*p* = 0.315).

Administration of antibiotics was not significantly associated with the development or prevention of surgical site infection in any category (no antibiotics, pre-operative antibiotics, peri-operative antibiotics, and post-operative antibiotics) (Table 1). Forty-six (20.4%) patients did not receive antibiotics of any kind, of which, five (10.9%) patients developed an infection. Twenty-six (11.5%) patients received pre-operative antibiotics, three (11.5%) of which developed an infection. One hundred forty-three (63.3%) patients received peri-operative antibiotics. Of those patients which received peri-operative antibiotics 10 (7%) developed infection. Post-operative antibiotics were prescribed in 95 (42.0%) patients, of which six (6.3%) developed an infection.

**Table 1:** Factors Associated with the Development of Surgical Site Infection (SSI).

**Risk Factors**	**No. dogs in category**	**No. (%) of dogs that developed SSI**	**OR (95% CI)**	***p*-value**
Comorbidities				
Endocrinopathy	2	1 (50.0)	Reference	
NSAIDS / Immunosuppressive	13	1 (7.7)	0.08 (0.00–2.60)	0.157
Time under anesthesia				0.048
0–4 h	100	7 (7.0)	Reference	
4–6 h	176	4 (2.3)	0.60 (0.17–2.13)	
>6 h	31	6 (19.4)	3.19 (0.98–10.3)	
Tumor type				0.718
Odontogenic	74	6 (8.1)	Reference	
Malignant	139	11 (7.9)	0.97 (0.35–2.75)	
Undetermined	3	0 (0)	NA	
Mixed malignant & odontogenic tumors	3	0 (0)	NA	
Margins				
Tumor-free margins	156	12 (7.7)	Reference	
Incomplete excision	41	4 (9.8)	1.30 (0.40–4.26)	0.315
Location of surgery				
Sterile operating room	120	13 (10.8)	Reference	
Dental suite	106	4 (3.8)	0.32 (0.10–1.02)	0.055
Antibiotic administration				
Pre-operative (Yes)	26	3 (11.5)	1.73 (0.46–6.49)	0.414
Peri-operative (Yes)	143	10 (7)	0.82 (0.30–2.23)	0.693
Post-operative (Yes)	95	6 (6.3)	0.74 (0.26–2.06)	0.559
Any antibiotics (Yes)	180	12 (6.7)	0.59 (0.20–1.76)	0.340

One-hundred six procedures were performed in a non-sterile dental suite, four of which (3.8%) developed an infection. The remaining 120 surgeries were performed in a sterile operating room, 13 of which (10.8%) developed an infection. Those procedures that were performed in a dental suite were less likely to develop surgical site infections as compared to those that were performed in a sterile operating room; a difference that approached but did not meet the benchmark for statistical significance (OR: [95% CI]: 0.32 [0.10–1.02]; *p* = 0.055). Table 1 provides a summary of the results of this statistical analysis.

## Discussion

The main objective of this report was to identify the incidence of surgical site infection within a population of dogs which underwent oncologic oromaxillofacial surgery. The reported results identified a 7.5% incidence of surgical site infection within the study population. This finding is consistent with previous reports for contaminated surgical procedures (3, 16). Additional objectives of this study were to identify risk factors associated with the incidence of infection including signalment, tumor type, use of peri-operative antibiotics, time under anesthesia, location of surgical procedure, specific comorbidities, and surgical margins obtained. Anesthetic events lasting greater than 6 h were significantly associated with development of infection while the remainder of the investigated risk factors had no significant effect on the incidence of surgical site infection.

Regarding use of antibiotics as a risk factor for development of surgical site infection, there was no significant difference in the incidence of infection between patients that did or did not receive any antibiotic treatment. Additionally, no significant difference in the incidence of infection was noted based on the timing of antibiotic administration (pre-, peri-, or post-operative). This finding suggests that administration of antibiotics for oromaxillofacial oncologic surgery, may not be routinely indicated for prevention of surgical site infection, despite the contaminated nature of these procedures.

In support of this finding, a previous study including 1,255 dogs and cats that underwent a variety of surgical procedures, not limited to the maxillofacial region, concluded that the degree of contamination was not useful in predicting surgical site infection rates (4). Surgical site infection rate following oromaxillofacial surgery has infrequently been reported previously in veterinary literature. A recent study investigating intraoperative and post-operative complications of maxillectomy surgery in 193 dogs, reported an infection rate of 7.9%, which is similar to the findings reported here (16). A second study evaluating a technique to prevent mandibular drift after mandibulectomy in 18 dogs reported a 0% infection rate (23). The authors of that study suggested that the low infection rate was due to use of a chlorhexidine oral rinse and professional dental cleaning prior to surgery and careful attention to surgical technique throughout the procedure. The low number of patients in the study may have also contributed to this lower infection rate. A third study reporting long-term follow up on 61 dogs which underwent maxillectomy reported only 1/61 (1.6%) patients developed surgical site infection (24). Antibiotic protocols were not considered in the aforementioned studies.

There is a larger body of literature in the human medical field regarding use of antibiotics during oromaxillofacial procedures (25–28). The human medical literature generally supports the use of peri-operative antibiotics for these surgeries, despite conflicting evidence of its efficacy. A systemic review of human head and neck surgery was performed with the goal to identify evidence regarding use of prophylactic antibiotic therapy (26). The authors concluded that there is minimal high-level evidence on this topic and that “no clear benefit of antibiotic prophylaxis” was identified. A second systematic literature review of 80 manuscripts regarding oral and maxillofacial surgery in humans concluded that peri-operative, but not post-operative, antibiotics can reduce the risk of surgical site infection in clean-contaminated oncologic surgery (27). The authors stated that use of peri-operative antibiotics is of “fundamental importance in maxillofacial surgery”. A third review article reporting antibiotic prophylaxis protocols in orthognathic surgery, implant surgery, and wisdom tooth extraction, recommended a pre-operative dose of antibiotic for all procedures (25). However, the authors admit there is little evidence on this topic and suggest that surgeons use their clinical judgement on a case-by-case basis to determine need for prophylactic antibiotic therapy.

Regrettably, the use of prophylactic antibiotic therapy may be driven by routine protocols that are not supported by evidence or may be driven by defensive medical decisions rather than robust evidence (15, 26). Although concern for antibiotic resistance is a driving force against the recommendation for prophylactic antibiotic therapy, there is evidence in the human literature to suggest that use of prophylactic, peri-operative antibiotics is unlikely to contribute to resistant antibiotic infections in individual patients. In a retrospective cohort study including adults who underwent a variety of elective surgical procedures, peri-operative prophylactic antibiotic therapy was not associated with risk for postoperative antibiotic-resistant infections (29). However, this does not address the significant risk of antibiotic resistance as it pertains to its effect on broader public health.

Both surgical time and time under anesthesia have previously been identified as significant risk factors for development of surgical site infection (2, 12). A retrospective study of 777 dogs and cats concluded that a significant risk factor for infection was time under anesthesia. This finding was independent of the surgical time (13). This is in direct contrast to our findings that increased anesthesia time, when considered as a whole population, did not contribute to the incidence of post-operative infection. However, our results did indicate that patients anesthetized for greater than 6 h were significantly more likely to develop surgical site infections as compared to patients anesthetized for 4–6 h but not significantly more likely to develop infections when compared to patients anesthetized for <4 h. These findings may be due to a sample size bias related to the distribution of patients across the three defined anesthetic time periods. Alternatively, these findings support the idea that development of surgical site infection is multifactorial and cannot be predicted by a single risk factor.

Our finding that patients anesthetized for the longest periods were most likely to develop infection is consistent with previous reports (13). Increased time under anesthesia may be associated with longer surgical time, more anesthetic complications and increased drug administration, all of which may contribute to incidence of infection (2, 4, 13, 30, 31). Additionally, longer anesthetic times are necessary for large excisions with potentially more tissue handling and highly technique sensitive surgeries. We attempted to include the surgical time as a variable in the present study, however, the surgical time was not accurately reported in many of these cases and, therefore, could not be considered. The wide range of time under general anesthesia reported here (1.25–10 h) can be explained by the various surgical procedures that were included in this analysis.

Those procedures which were performed in a dental suite were less likely to develop surgical site infection as compared to procedures performed in a sterile operating room. Although not statistically significant, the *p* value was 0.055. The authors consider three potential explanations for this finding. First, the patients treated in the dental suite are, anecdotally, more likely to have a pre-operative chlorhexidine rinse and/or professional dental scaling and polishing performed prior to the surgical procedure resulting in reduced bacterial load in the oral cavity (15, 23, 32, 33). Reducing the bacterial load within the oral cavity prior to starting an oncologic surgery would theoretically decrease the degree of contamination of the procedure. Many references (15, 32, 34) recommend dental scaling and / or antibiotic lavage prior to performing surgery which involves the oral cavity; however, no evidence is available to support this as a possible cause of reduced surgical site infections. Second, at this institution, oromaxillofacial surgery performed in the dental suite is generally for treatment of patients with lower ASA status and tumors requiring less technically sensitive procedures, smaller en bloc excisions generally in more rostral locations and, therefore, shortened anesthetic procedures. Smaller en bloc excisions would also likely result in less surgical time and therefore less handling of tissues, less suture material used, and reduced area of surgical healing, all of which have been theorized as potential risk factors to increase risk of infection (4). Third, it is standard practice at this institution to use sterile draping and instrument technique when performing surgery in the dental suite. The primary surgeon in the dental suite does not perform a surgical scrub, but is adorned with sterile gloves and a surgical cap. The assisting personnel are not routinely outfitted with traditional operating room attire, however, as they would be when assisting in the operating room.

Patients with endocrinopathies have previously been reported to have increased risk of post-operative infection (12). Only two patients were described as having endocrinopathies in this study population. This prevented any relevant statistical analysis of the association between endocrinopathies and the development of surgical site infection. The low number of patients with endocrinopathies is likely due to the retrospective nature of data collection and the tertiary nature of this institution where many patients present with incomplete referral records and chronic illnesses are not generally managed by the surgical teams.

Limitations of this study may have contributed to the lack of statistically significant findings in this study. First, statistical significance may not have been identified based on the sample size. Second, a wide variety of surgical procedures were included in this investigation under the inclusion of oromaxillofacial oncologic surgery. Further differentiation between specific surgical procedures or differing orofacial regions may reveal novel results. Type II error, lack of statistical significance when in fact there is an association between the variable and outcome, is a third explanation for the lack of significant findings here. Conversely, it is always possible that type I error, giving significance to a variable when the association merely occurred by chance, resulted in untrustworthy significance in this data. The retrospective nature of this report also has inherent limitations. There were a few variables that have previously been identified as potential risk factors for surgical site infection, including the ASA classification status, surgical time, and anesthetic complications (such as hypotension) that were not considered in this analysis due to insufficient detail in the medical records. Our inclusion criteria required an examination at this institution or with the referring veterinarian at least 7 days following the surgery. This institution generally recommended post-surgical recheck appointments 7–21 days following surgical procedure and patients may have been examined after that time and still been included in this analysis. If any patient had a surgical site infection that occurred after the post-surgical recheck or prior to the post-surgical recheck and was self-resolved by the time of the post-surgical assessment, this may have under-reported the incidence of surgical site infection.

During the medical record review, numerous patients were prescribed antibiotic therapy at their post-operative recheck appointment (generally 7–21 days following the surgical procedure) without mention of evidence of a surgical site infection, physical exam findings describing purulent discharge, heat, swelling, pain, or a positive bacterial culture. Therefore, despite the antimicrobial prescription that was provided, there was no evidence in the medical record that a surgical site infection was present. It is unclear why these patients were prescribed antibiotics but for the purposes of this study, we determined it prudent to presume the reason to be prophylactic in nature (e.g., after dehiscence of the surgical site). This assumption may lead to an under-representation of the incidence of infection in the sample population.

In conclusion, use of antibiotics during oromaxillofacial oncologic surgery did not result in a decreased incidence of surgical site infection in dogs. Prophylactic antibiotic therapy may not be routinely necessary when surgically treating oromaxillofacial tumors. This report identified that those anesthetic events lasting greater than 6 h had a significantly increased incidence of surgical site infection and prophylactic antibiotic therapy should be considered in oncologic oromaxillofacial surgeries which require anesthetic time of 6 h or longer. Performing oromaxillofacial oncologic surgery within a non-sterile dental suite did not increase the risk of infection within this study population. However, the location of surgery as a risk factor for surgical site infection should be further evaluated, ideally in a prospective manner.

## Data Availability Statement

The raw data supporting the conclusions of this article will be made available by the authors, without undue reservation.

## Author Contributions

BR: conception and design of project, acquisition, analysis and interpretation of data, manuscript drafting, and revision. KM: acquisition of data, manuscript revision. SH: design of project, data analysis, and manuscript revision. JS: conception and design of project, analysis and interpretation of data, manuscript drafting, and revision. All authors listed have made a substantial, direct and intellectual contribution to the work, and approved it for publication.

## Funding

This project was supported by funding from the Institutional Clinical and Translational Science Award UL1 TR002373.

## Conflict of Interest

The authors declare that the research was conducted in the absence of any commercial or financial relationships that could be construed as a potential conflict of interest.

## Publisher's Note

All claims expressed in this article are solely those of the authors and do not necessarily represent those of their affiliated organizations, or those of the publisher, the editors and the reviewers. Any product that may be evaluated in this article, or claim that may be made by its manufacturer, is not guaranteed or endorsed by the publisher.
